# Poly[(μ_3_-5-bromo­nicotinato)(5-bromo­nicotinato)copper(II)]

**DOI:** 10.1107/S1600536809038331

**Published:** 2009-09-30

**Authors:** Jun Yang, Hong-Ji Chen

**Affiliations:** a Department of Materials Science and Engineering, Jinan University, Guangzhou 510632, People’s Republic of China

## Abstract

The title coordination polymer, [Cu(C_6_H_3_BrNO_2_)_2_]_*n*_, is composed of two structurally similar two-dimensional coordination polymers (twin layers). Both of them have the same chemical composition but they display different bond lengths and angles. In each layer, two N atoms and four carboxyl­ate O atoms from the bridging 5-bromo­nicotinate ligands and four carboxyl­ate O atoms from the terminal 5-bromo­nicotinate ligands bind to two Cu^II^ atoms to form a dinuclear paddle-wheel-like pattern. Adjacent paddle wheels are further linked by bridging 5-bromo­nicotinate groups to generate a two-dimensional coordination polymer; neighboring twin-like layers are finally stacked through π–π stacking interactions between adjacent pyridine rings [perpendicular distance of 3.626 (2) Å] in a ‘sandwich’ manner, thus generating a three-dimensional supra­molecular structure.

## Related literature

For related literature on paddle-wheel secondary building units, see: Chen *et al.* (2006 ▶); Xue *et al.* (2007 ▶); Striegler & Dittel (2003 ▶); Ma & Moulton (2007 ▶); Banerjee *et al.* (2008 ▶); Saravanakumar *et al.* (2004 ▶). For similar structures, see: Yakovenko *et al.* (2009 ▶); Xue *et al.* (2007 ▶). For τ distortions of coordination polyhedra, see: Addison & Rao (1984 ▶).
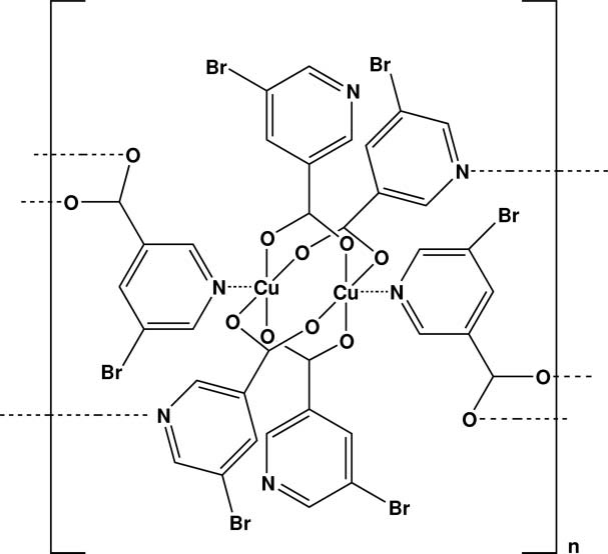


## Experimental

### 

#### Crystal data

[Cu(C_6_H_3_BrNO_2_)_2_]
*M* *_r_* = 465.55
Monoclinic, 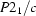

*a* = 21.542 (4) Å
*b* = 11.746 (2) Å
*c* = 12.271 (2) Å
β = 104.31 (3)°
*V* = 3008.6 (9) Å^3^
*Z* = 8
Mo *K*α radiation
μ = 6.78 mm^−1^
*T* = 173 K
0.33 × 0.31 × 0.23 mm

#### Data collection

Bruker SMART CCD area-detector diffractometer
Absorption correction: multi-scan (*SADABS*; Sheldrick, 2004 ▶) *T*_min_ = 0.213, *T*_max_ = 0.305
15126 measured reflections
6502 independent reflections
4904 reflections with *I* > 2σ(*I*)
*R*_int_ = 0.031

#### Refinement

*R*[*F* ^2^ > 2σ(*F* ^2^)] = 0.034
*wR*(*F* ^2^) = 0.085
*S* = 1.03
6502 reflections
379 parameters
H-atom parameters constrained
Δρ_max_ = 1.40 e Å^−3^
Δρ_min_ = −1.34 e Å^−3^

### 

Data collection: *SMART* (Bruker, 1998 ▶); cell refinement: *SAINT* (Bruker, 1998 ▶); data reduction: *SAINT* (Bruker, 1998 ▶); program(s) used to solve structure: *SHELXS97* (Sheldrick, 2008 ▶); program(s) used to refine structure: *SHELXL97* (Sheldrick, 2008 ▶); molecular graphics: *DIAMOND* (Brandenburg, 2005 ▶); software used to prepare material for publication: *SHELXTL* (Sheldrick, 2008 ▶).

## Supplementary Material

Crystal structure: contains datablocks I, global. DOI: 10.1107/S1600536809038331/bg2298sup1.cif

Structure factors: contains datablocks I. DOI: 10.1107/S1600536809038331/bg2298Isup2.hkl

Additional supplementary materials:  crystallographic information; 3D view; checkCIF report

## Figures and Tables

**Fig. 1.**
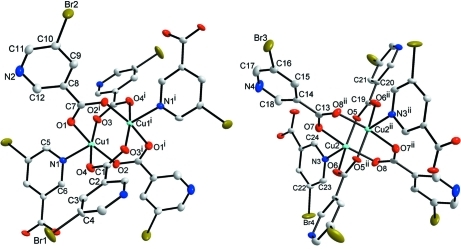
The coordination environment of Cu1 and Cu2 atoms in the title compound, showing displacement ellipsoids at the 35% probability level. Symmetry operator: i = 1-x, 1-y, 1-z; ii = 2-x, 1-y, -z;

**Fig. 2.**
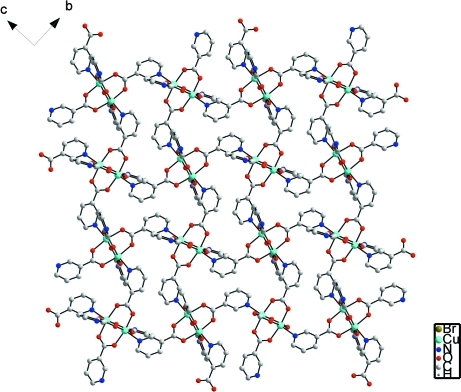
A (100) view of the structure showing a twin two-dimensional coordination polymer, representing either layer A or layer B (Hydrogen and Bromine atoms are omitted for clarity).

**Fig. 3.**
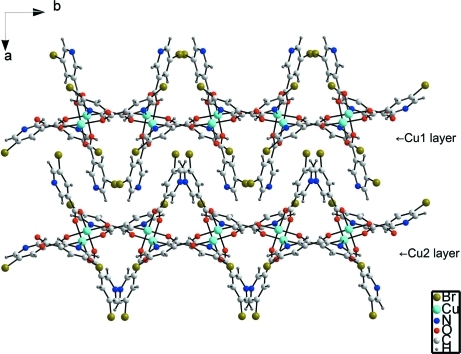
A [001] view of the packing pattern of the twin layers.

## supplementary crystallographic information

### Comment

Paddle-wheel secondary building units (SBUs) *M*_2_(RCOO)_4_ are useful building blocks for constructing complexes and 1D to 3D coordination polymers through bifunctional ligands. Research interest in these compounds with copper(II) paddle-wheel SBUs come from their structural diversity (Chen, *et al.*, 2006; Xue *et al.*, 2007) and potential applications in supramolecular medicinal chemistry (Ma & Moulton 2007), sugar discrimination (Striegler & Dittel 2003), molecular magnets (Banerjee *et al.*, 2008; Saravanakumar *et al.*, 2004; Yakovenko, *et al.*, 2009), respectively. However, most reported Cu(II) complexes with paddle-whell secondary building units are constructed by mixed-ligands, either two kinds of organic ligands or one organic ligand and water molecules (Ma & Moulton 2007). 5-Bromonicotinic acid is a bifunctional ligand with two carboxylic oxygen atoms and one pyridyl nitrogen atom, and it can be coordinated to a metal centre in a variety of ways to create structural diversity. Here, we report a new two-dimentional coordination polymer with Cu(II) paddle-wheel SBUs formed from the organic ligand 5-Bromonicotinic acid and Cu(II) ions.

The title coordination polymer, twin-poly[copper(II) (η-*N*,*O*,*O*-5-bromonicotinato)(η-*O*,*O*-5-bromonicotinato)] (I), contains two similar, independent groups in the asymmetric unit consisting of two copper atoms (Cu1 and Cu2) and four 5-bromonicotinato ligands each. The coordination enviroments of the two copper atoms present a nearly perfect [CuO_4_N] square pyramid geometry, characterized by τ factors (indicative of the distortion degree of such a coordination sphere, Addison & Rao, 1984), of 0.01 for Cu1 and 0.003 for Cu2. The bond lengths and angles around Cu1 and Cu2 are standard for a Jahn-Teller active square pyramidal Cu^2+^ ion, with basal Cu—O bond lengths ranging from 1.957 (3) Å to 1.979 (3) Å (1.952 (3)–1.976 (3) Å ) and a longer axial bond distance to the the ligand nitrogen atom of 2.150 (3) Å (2.164 (3) Å) for Cu1 (Cu2), respectively (Fig. 1).

As previously stated, each copper atom (Cu1 or Cu2) is located in a penta-coordinated geometry and is bonded by four oxygen atoms from carboxylate anions and one nitrogen atom from the axial ligand, which can also be considered as a molecular square when viewed along the axial direction. When four carboxylate groups of the ligands bridge to two asymmetric copper atoms in a *syn*-*syn* manner, a dinuclear paddle-wheel pattern is formed, with Cu—Cu distances of 2.6355 (10) Å for Cu1 and 2.6423 (10) Å for Cu2. These values are similar to those in the paddle-wheel copper(II) complex reported in Yakovenko, *et al.* 2009, but shorter than the corresponding ones in Xue *et al.* 2007.

When adjacent Cu1 paddle-wheels are bridged by the η-*N*,*O*,*O*-5-bromonicotinato groups, a layer motif (hereafter "A", [Cu1(η-*N*,*O*,*O*-5-bromonicotinato)(η-*O*,*O*-5-bromonicotinato)]_n )_, is formed along (100). Similarly, those resulting from Cu2 generate another layer motif ( "B", [Cu2(η-*N*,*O*,*O*-5-bromonicotinato)(η-*O*,*O*-5-bromonicotinato)]_n_) which lies parallel to the former (Fig. 2). Finally, both A and B layers contact along the c axial direction generating a new, twin-like coordination polymer (Fig. 3). Neighboring twin-like layers are further stacked via van der Waals interactions in a sandwich way extending the packing into a three-dimensional supramolecular structure. No significant hydrogen bonds were found in the crystal structure.

### Experimental

Copper nitrate trihydrate (0.4 mmol, 0.0966 g) in 10 ml water and 5-Bromonicotinic acid (0.4 mmol, 0.0808 g) were sealed in a Teflon-line autoclave and heated to 433 K for 72 h, after which the mixture was cooled down to room temperatureat at a rate of 5 K per hour. Blue single crystals suitable for *x*-ray crystallography analysis were obtained with a yield of 46 percent. IR (cm^-1^, KBr): 3447*m*, 3057w, 1639vs, 1557 s, 1443vs, 1393vs, 1292 s, 1238w, 1178w, 1143*m*, 1024*m*, 904w, 877*m*, 781 s, 747vs, 685*m*, 497 s.

### Refinement

Hydrogen atoms of the 5-bromonicotinato groups were placed at calculated positions and allowed to ride on their respective parent atoms with C—H distances in the range of 0.96–0.98 Å. The structure contains solvent accessible voids of 61 Å^3^ in its lattice, slightly larger than the threshold voids (40 Å^3^) for general accommodable water molecules. However, no trace of unaccounted for electron density could be detected in the difference maps, for what it can be safely assumed that any eventually trapped solvato molecules would not occupy stable positions.

### Crystal data

**Table:**

[Cu(C_6_H_3_BrNO_2_)_2_]	*Z* = 8
*M**_r_* = 465.55	*F*(000) = 1784
Monoclinic, *P*2_1_/*c*	*D*_x_ = 2.056 Mg m^−^^3^
Hall symbol: -P 2ybc	Mo *K*α radiation, λ = 0.71073 Å
*a* = 21.542 (4) Å	θ = 2.4–27.0°
*b* = 11.746 (2) Å	µ = 6.78 mm^−^^1^
*c* = 12.271 (2) Å	*T* = 173 K
β = 104.31 (3)°	Block, blue
*V* = 3008.6 (9) Å^3^	0.33 × 0.31 × 0.23 mm

### Data collection

**Table:**

Bruker SMART CCD area-detector diffractometer	6502 independent reflections
Radiation source: fine-focus sealed tube	4904 reflections with *I* > 2σ(*I*)
graphite	*R*_int_ = 0.031
φ and ω scans	θ_max_ = 27.0°, θ_min_ = 2.0°
Absorption correction: multi-scan (*SADABS*; Sheldrick, 2004)	*h* = −27→15
*T*_min_ = 0.213, *T*_max_ = 0.305	*k* = −12→15
15126 measured reflections	*l* = −15→15

### Refinement

**Table:**

Refinement on *F*^2^	Primary atom site location: structure-invariant direct methods
Least-squares matrix: full	Secondary atom site location: difference Fourier map
*R*[*F*^2^ > 2σ(*F*^2^)] = 0.034	Hydrogen site location: inferred from neighbouring sites
*wR*(*F*^2^) = 0.085	H-atom parameters constrained
*S* = 1.02	*w* = 1/[σ^2^(*F*_o_^2^) + (0.0329*P*)^2^ + 6.737*P*] where *P* = (*F*_o_^2^ + 2*F*_c_^2^)/3
6502 reflections	(Δ/σ)_max_ = 0.002
379 parameters	Δρ_max_ = 1.40 e Å^−^^3^
0 restraints	Δρ_min_ = −1.33 e Å^−^^3^

### Special details

**Table:**

Geometry. All e.s.d.'s (except the e.s.d. in the dihedral angle between two l.s. planes) are estimated using the full covariance matrix. The cell e.s.d.'s are taken into account individually in the estimation of e.s.d.'s in distances, angles and torsion angles; correlations between e.s.d.'s in cell parameters are only used when they are defined by crystal symmetry. An approximate (isotropic) treatment of cell e.s.d.'s is used for estimating e.s.d.'s involving l.s. planes.
Refinement. Refinement of *F*^2^ against ALL reflections. The weighted *R*-factor *wR* and goodness of fit *S* are based on *F*^2^, conventional *R*-factors *R* are based on *F*, with *F* set to zero for negative *F*^2^. The threshold expression of *F*^2^ > σ(*F*^2^) is used only for calculating *R*-factors(gt) *etc*. and is not relevant to the choice of reflections for refinement. *R*-factors based on *F*^2^ are statistically about twice as large as those based on *F*, and *R*- factors based on ALL data will be even larger.

### Fractional atomic coordinates and isotropic or equivalent isotropic displacement parameters (Å^2^)

**Table:**

	*x*	*y*	*z*	*U*_iso_*/*U*_eq_	
Br1	0.36048 (3)	1.10765 (4)	0.45763 (4)	0.04098 (14)	
Br2	0.22167 (2)	0.26934 (6)	0.04959 (4)	0.04929 (17)	
Br3	0.65085 (2)	0.30249 (5)	−0.19347 (4)	0.04179 (15)	
Br4	1.13503 (3)	0.60918 (5)	0.58445 (4)	0.05030 (18)	
Cu1	0.48131 (2)	0.52244 (4)	0.59219 (4)	0.01204 (10)	
Cu2	1.02261 (2)	0.48099 (4)	0.10871 (4)	0.01222 (10)	
N1	0.46040 (15)	0.5444 (3)	0.7534 (3)	0.0155 (7)	
N2	0.20763 (17)	0.4113 (4)	0.3472 (3)	0.0318 (9)	
N3	1.04924 (15)	0.4524 (3)	0.2885 (3)	0.0158 (7)	
N4	0.76951 (19)	0.2695 (4)	0.1228 (3)	0.0389 (10)	
O1	0.39446 (13)	0.4902 (3)	0.5031 (2)	0.0261 (7)	
O2	0.57262 (12)	0.5555 (3)	0.6502 (2)	0.0223 (6)	
O3	0.49764 (14)	0.3579 (2)	0.6129 (2)	0.0223 (6)	
O4	0.46875 (13)	0.6827 (2)	0.5418 (2)	0.0207 (6)	
O5	1.04325 (13)	0.3262 (2)	0.0652 (2)	0.0179 (6)	
O6	0.99585 (14)	0.6387 (2)	0.1185 (2)	0.0247 (7)	
O7	0.93424 (13)	0.4281 (3)	0.0966 (2)	0.0231 (6)	
O8	1.10483 (13)	0.5363 (2)	0.0872 (2)	0.0239 (7)	
C1	0.48052 (18)	0.7079 (3)	0.4500 (3)	0.0157 (8)	
C2	0.46378 (17)	0.8249 (3)	0.4044 (3)	0.0128 (7)	
C3	0.42926 (18)	0.9003 (3)	0.4536 (3)	0.0176 (8)	
H3B	0.4191	0.8821	0.5234	0.080*	
C4	0.41056 (19)	1.0027 (3)	0.4004 (3)	0.0205 (9)	
C5	0.42629 (19)	0.4721 (3)	0.7994 (3)	0.0198 (8)	
H5A	0.4144	0.4051	0.7657	0.050*	
C6	0.47896 (17)	0.6433 (3)	0.8054 (3)	0.0153 (8)	
H6A	0.5027	0.6896	0.7725	0.050*	
C7	0.38534 (18)	0.4560 (3)	0.4041 (3)	0.0169 (8)	
C8	0.31838 (17)	0.4236 (3)	0.3453 (3)	0.0175 (8)	
C9	0.30550 (19)	0.3730 (4)	0.2396 (4)	0.0241 (9)	
H9A	0.3388	0.3606	0.2015	0.080*	
C10	0.24266 (19)	0.3414 (4)	0.1909 (3)	0.0241 (9)	
C11	0.19555 (19)	0.3611 (4)	0.2464 (4)	0.0249 (9)	
H11A	0.1525	0.3381	0.2115	0.080*	
C12	0.2679 (2)	0.4414 (4)	0.3946 (4)	0.0260 (9)	
H12A	0.2769	0.4772	0.4672	0.080*	
C13	0.89075 (18)	0.4275 (3)	0.0067 (3)	0.0176 (8)	
C14	0.82826 (18)	0.3749 (3)	0.0129 (3)	0.0177 (8)	
C15	0.77755 (19)	0.3685 (3)	−0.0817 (3)	0.0208 (9)	
H15A	0.7803	0.4012	−0.1520	0.080*	
C16	0.72308 (18)	0.3128 (4)	−0.0705 (3)	0.0226 (9)	
C17	0.7203 (2)	0.2648 (4)	0.0305 (4)	0.0325 (11)	
H17A	0.6817	0.2266	0.0354	0.080*	
C18	0.8221 (2)	0.3253 (4)	0.1122 (4)	0.0301 (10)	
H18A	0.8573	0.3313	0.1774	0.080*	
C19	1.02976 (18)	0.2986 (3)	−0.0370 (3)	0.0152 (8)	
C20	1.04662 (17)	0.1814 (3)	−0.0668 (3)	0.0132 (7)	
C21	1.07623 (18)	0.1035 (3)	0.0149 (3)	0.0154 (8)	
H21A	1.0841	0.1230	0.0950	0.050*	
C22	1.09175 (19)	0.5010 (3)	0.4790 (3)	0.0191 (8)	
C23	1.07778 (18)	0.5271 (3)	0.3658 (3)	0.0182 (8)	
H23A	1.0892	0.6007	0.3428	0.080*	
C24	1.03400 (17)	0.3506 (3)	0.3220 (3)	0.0151 (8)	
H24A	1.0114	0.2988	0.2626	0.050*	

### Atomic displacement parameters (Å^2^)

**Table:**

	*U*^11^	*U*^22^	*U*^33^	*U*^12^	*U*^13^	*U*^23^
Br1	0.0617 (3)	0.0352 (3)	0.0350 (3)	0.0291 (2)	0.0292 (2)	0.0086 (2)
Br2	0.0306 (3)	0.0809 (4)	0.0360 (3)	−0.0217 (3)	0.0076 (2)	−0.0310 (3)
Br3	0.0203 (2)	0.0719 (4)	0.0300 (3)	−0.0167 (2)	0.00016 (18)	−0.0005 (2)
Br4	0.0942 (5)	0.0338 (3)	0.0177 (2)	−0.0389 (3)	0.0040 (2)	−0.0080 (2)
Cu1	0.0157 (2)	0.0104 (2)	0.0101 (2)	−0.00042 (17)	0.00348 (17)	−0.00025 (17)
Cu2	0.0160 (2)	0.0096 (2)	0.0094 (2)	−0.00019 (17)	0.00013 (17)	−0.00022 (17)
N1	0.0182 (16)	0.0147 (18)	0.0131 (15)	−0.0002 (12)	0.0029 (13)	−0.0016 (13)
N2	0.0186 (19)	0.050 (3)	0.026 (2)	0.0006 (17)	0.0042 (15)	−0.0037 (18)
N3	0.0203 (17)	0.0119 (17)	0.0147 (16)	−0.0012 (12)	0.0031 (13)	0.0012 (12)
N4	0.032 (2)	0.060 (3)	0.027 (2)	−0.011 (2)	0.0113 (17)	0.008 (2)
O1	0.0174 (15)	0.0376 (19)	0.0224 (15)	−0.0036 (12)	0.0032 (12)	−0.0094 (13)
O2	0.0145 (14)	0.0315 (17)	0.0205 (15)	−0.0023 (12)	0.0034 (11)	−0.0019 (12)
O3	0.0369 (17)	0.0149 (15)	0.0186 (14)	0.0039 (12)	0.0136 (13)	0.0021 (12)
O4	0.0353 (17)	0.0144 (15)	0.0163 (14)	0.0052 (12)	0.0137 (12)	0.0061 (11)
O5	0.0270 (15)	0.0137 (14)	0.0112 (13)	0.0033 (11)	0.0016 (11)	−0.0041 (11)
O6	0.0450 (18)	0.0119 (15)	0.0146 (14)	0.0080 (13)	0.0023 (13)	0.0011 (11)
O7	0.0167 (14)	0.0336 (18)	0.0182 (15)	−0.0019 (12)	0.0032 (11)	−0.0022 (13)
O8	0.0197 (15)	0.0284 (18)	0.0199 (15)	−0.0079 (12)	−0.0018 (11)	0.0085 (12)
C1	0.0186 (19)	0.012 (2)	0.0155 (19)	−0.0014 (15)	0.0026 (15)	0.0005 (15)
C2	0.0133 (18)	0.015 (2)	0.0089 (17)	−0.0015 (14)	0.0002 (13)	0.0002 (14)
C3	0.020 (2)	0.017 (2)	0.0148 (19)	−0.0020 (15)	0.0037 (15)	0.0015 (16)
C4	0.026 (2)	0.017 (2)	0.022 (2)	0.0073 (16)	0.0133 (17)	−0.0017 (16)
C5	0.027 (2)	0.015 (2)	0.018 (2)	−0.0065 (16)	0.0073 (16)	−0.0065 (16)
C6	0.0158 (19)	0.013 (2)	0.0171 (19)	0.0023 (14)	0.0038 (15)	0.0000 (15)
C7	0.019 (2)	0.011 (2)	0.020 (2)	0.0009 (15)	0.0044 (16)	0.0032 (15)
C8	0.0137 (19)	0.018 (2)	0.019 (2)	0.0009 (15)	0.0003 (15)	0.0000 (16)
C9	0.019 (2)	0.029 (3)	0.024 (2)	−0.0038 (17)	0.0038 (17)	−0.0036 (18)
C10	0.020 (2)	0.030 (3)	0.020 (2)	−0.0046 (17)	0.0007 (17)	−0.0026 (18)
C11	0.017 (2)	0.031 (3)	0.025 (2)	−0.0019 (17)	0.0021 (17)	0.0012 (19)
C12	0.021 (2)	0.034 (3)	0.021 (2)	0.0016 (18)	0.0025 (17)	−0.0024 (19)
C13	0.020 (2)	0.0084 (19)	0.026 (2)	0.0012 (15)	0.0073 (17)	−0.0038 (16)
C14	0.020 (2)	0.013 (2)	0.020 (2)	−0.0019 (15)	0.0047 (16)	−0.0020 (16)
C15	0.021 (2)	0.016 (2)	0.025 (2)	−0.0002 (16)	0.0051 (17)	−0.0003 (17)
C16	0.016 (2)	0.027 (2)	0.023 (2)	−0.0009 (17)	0.0006 (16)	−0.0026 (18)
C17	0.025 (2)	0.041 (3)	0.033 (3)	−0.007 (2)	0.011 (2)	0.005 (2)
C18	0.025 (2)	0.046 (3)	0.018 (2)	−0.005 (2)	0.0044 (18)	0.001 (2)
C19	0.0194 (19)	0.0101 (19)	0.0167 (19)	−0.0020 (15)	0.0055 (15)	−0.0018 (15)
C20	0.0160 (18)	0.0079 (19)	0.0165 (19)	0.0006 (14)	0.0052 (14)	−0.0035 (14)
C21	0.022 (2)	0.015 (2)	0.0075 (17)	0.0018 (15)	0.0015 (14)	−0.0012 (14)
C22	0.028 (2)	0.016 (2)	0.0123 (18)	−0.0059 (16)	0.0021 (16)	−0.0051 (15)
C23	0.023 (2)	0.013 (2)	0.018 (2)	−0.0047 (15)	0.0039 (16)	−0.0005 (16)
C24	0.0185 (19)	0.0119 (19)	0.0139 (18)	0.0004 (15)	0.0019 (15)	0.0010 (15)

### Geometric parameters (Å, °)

**Table:**

Br1—C4	1.885 (4)	C3—C4	1.380 (5)
Br2—C10	1.881 (4)	C3—H3B	0.9600
Br3—C16	1.885 (4)	C4—C5^iii^	1.394 (5)
Br4—C22	1.887 (4)	C5—C4^iv^	1.394 (5)
Cu1—O1	1.957 (3)	C5—H5A	0.8971
Cu1—O2	1.958 (3)	C6—C2^iv^	1.386 (5)
Cu1—O3	1.969 (3)	C6—H6A	0.9065
Cu1—O4	1.979 (3)	C7—O2^i^	1.257 (5)
Cu1—N1	2.150 (3)	C7—C8	1.494 (5)
Cu1—Cu1^i^	2.6355 (10)	C8—C12	1.385 (6)
Cu2—O6	1.952 (3)	C8—C9	1.390 (6)
Cu2—O8	1.965 (3)	C9—C10	1.389 (5)
Cu2—O7	1.973 (3)	C9—H9A	0.9601
Cu2—O5	1.976 (3)	C10—C11	1.375 (6)
Cu2—N3	2.164 (3)	C11—H11A	0.9599
Cu2—Cu2^ii^	2.6423 (10)	C12—H12A	0.9601
N1—C5	1.336 (5)	C13—O8^ii^	1.254 (5)
N1—C6	1.338 (5)	C13—C14	1.500 (5)
N2—C12	1.333 (5)	C14—C15	1.385 (6)
N2—C11	1.337 (5)	C14—C18	1.386 (6)
N3—C23	1.327 (5)	C15—C16	1.379 (6)
N3—C24	1.332 (5)	C15—H15A	0.9600
N4—C18	1.343 (6)	C16—C17	1.376 (6)
N4—C17	1.347 (6)	C17—H17A	0.9601
O1—C7	1.247 (5)	C18—H18A	0.9600
O2—C7^i^	1.257 (5)	C19—O6^ii^	1.254 (4)
O3—C1^i^	1.263 (5)	C19—C20	1.492 (5)
O4—C1	1.250 (4)	C20—C24^v^	1.376 (5)
O5—C19	1.258 (4)	C20—C21	1.390 (5)
O6—C19^ii^	1.254 (4)	C21—C22^v^	1.373 (5)
O7—C13	1.258 (5)	C21—H21A	0.9821
O8—C13^ii^	1.254 (5)	C22—C21^vi^	1.373 (5)
C1—O3^i^	1.263 (5)	C22—C23	1.381 (5)
C1—C2	1.494 (5)	C23—H23A	0.9600
C2—C6^iii^	1.386 (5)	C24—C20^vi^	1.376 (5)
C2—C3	1.386 (5)	C24—H24A	0.9814
			
O1—Cu1—O2	167.78 (12)	N1—C5—H5A	119.1
O1—Cu1—O3	89.88 (13)	C4^iv^—C5—H5A	119.5
O2—Cu1—O3	90.95 (12)	N1—C6—C2^iv^	123.0 (4)
O1—Cu1—O4	88.36 (13)	N1—C6—H6A	116.4
O2—Cu1—O4	88.40 (12)	C2^iv^—C6—H6A	120.7
O3—Cu1—O4	168.51 (11)	O1—C7—O2^i^	126.1 (4)
O1—Cu1—N1	98.55 (12)	O1—C7—C8	117.0 (3)
O2—Cu1—N1	93.55 (12)	O2^i^—C7—C8	116.9 (3)
O3—Cu1—N1	94.02 (11)	C12—C8—C9	118.3 (4)
O4—Cu1—N1	97.47 (11)	C12—C8—C7	121.3 (4)
O1—Cu1—Cu1^i^	86.23 (9)	C9—C8—C7	120.4 (3)
O2—Cu1—Cu1^i^	81.90 (9)	C10—C9—C8	117.7 (4)
O3—Cu1—Cu1^i^	80.33 (8)	C10—C9—H9A	121.1
O4—Cu1—Cu1^i^	88.23 (8)	C8—C9—H9A	121.2
N1—Cu1—Cu1^i^	172.64 (9)	C11—C10—C9	120.1 (4)
O6—Cu2—O8	89.09 (13)	C11—C10—Br2	119.6 (3)
O6—Cu2—O7	90.52 (13)	C9—C10—Br2	120.3 (3)
O8—Cu2—O7	168.27 (11)	N2—C11—C10	122.3 (4)
O6—Cu2—O5	168.22 (11)	N2—C11—H11A	118.9
O8—Cu2—O5	89.85 (12)	C10—C11—H11A	118.8
O7—Cu2—O5	88.14 (12)	N2—C12—C8	123.7 (4)
O6—Cu2—N3	95.31 (12)	N2—C12—H12A	118.3
O8—Cu2—N3	99.66 (12)	C8—C12—H12A	118.0
O7—Cu2—N3	92.04 (12)	O8^ii^—C13—O7	126.4 (4)
O5—Cu2—N3	96.44 (11)	O8^ii^—C13—C14	117.3 (3)
O6—Cu2—Cu2^ii^	82.01 (8)	O7—C13—C14	116.2 (4)
O8—Cu2—Cu2^ii^	85.86 (9)	C15—C14—C18	119.1 (4)
O7—Cu2—Cu2^ii^	82.48 (9)	C15—C14—C13	120.8 (4)
O5—Cu2—Cu2^ii^	86.21 (8)	C18—C14—C13	120.0 (4)
N3—Cu2—Cu2^ii^	173.85 (9)	C16—C15—C14	117.2 (4)
C5—N1—C6	118.8 (3)	C16—C15—H15A	121.5
C5—N1—Cu1	125.1 (3)	C14—C15—H15A	121.4
C6—N1—Cu1	115.9 (2)	C17—C16—C15	120.8 (4)
C12—N2—C11	117.8 (4)	C17—C16—Br3	118.9 (3)
C23—N3—C24	118.6 (3)	C15—C16—Br3	120.3 (3)
C23—N3—Cu2	125.9 (3)	N4—C17—C16	122.7 (4)
C24—N3—Cu2	115.5 (2)	N4—C17—H17A	118.7
C18—N4—C17	116.5 (4)	C16—C17—H17A	118.6
C7—O1—Cu1	120.4 (3)	N4—C18—C14	123.8 (4)
C7^i^—O2—Cu1	125.2 (3)	N4—C18—H18A	117.9
C1^i^—O3—Cu1	127.1 (2)	C14—C18—H18A	118.3
C1—O4—Cu1	117.7 (2)	O6^ii^—C19—O5	126.2 (4)
C19—O5—Cu2	119.7 (2)	O6^ii^—C19—C20	115.6 (3)
C19^ii^—O6—Cu2	125.9 (3)	O5—C19—C20	118.2 (3)
C13—O7—Cu2	124.3 (3)	C24^v^—C20—C21	118.6 (3)
C13^ii^—O8—Cu2	120.8 (2)	C24^v^—C20—C19	119.6 (3)
O4—C1—O3^i^	126.5 (4)	C21—C20—C19	121.8 (3)
O4—C1—C2	118.1 (3)	C22^v^—C21—C20	117.5 (3)
O3^i^—C1—C2	115.2 (3)	C22^v^—C21—H21A	122.2
C6^iii^—C2—C3	118.6 (3)	C20—C21—H21A	120.3
C6^iii^—C2—C1	119.2 (3)	C21^vi^—C22—C23	120.7 (3)
C3—C2—C1	122.0 (3)	C21^vi^—C22—Br4	120.0 (3)
C4—C3—C2	118.4 (4)	C23—C22—Br4	119.2 (3)
C4—C3—H3B	120.8	N3—C23—C22	121.4 (4)
C2—C3—H3B	120.8	N3—C23—H23A	119.5
C3—C4—C5^iii^	119.9 (4)	C22—C23—H23A	119.2
C3—C4—Br1	121.4 (3)	N3—C24—C20^vi^	123.2 (3)
C5^iii^—C4—Br1	118.7 (3)	N3—C24—H24A	116.2
N1—C5—C4^iv^	121.4 (4)	C20^vi^—C24—H24A	120.5

Symmetry codes: (i) −*x*+1, −*y*+1, −*z*+1; (ii) −*x*+2, −*y*+1, −*z*; (iii) *x*, −*y*+3/2, *z*−1/2; (iv) *x*, −*y*+3/2, *z*+1/2; (v) *x*, −*y*+1/2, *z*−1/2; (vi) *x*, −*y*+1/2, *z*+1/2.
